# Integrating palliative care into national health systems in Africa: a multi–country intervention study

**DOI:** 10.7189/jogh.07.010419

**Published:** 2017-06

**Authors:** Liz Grant, Julia Downing, Emmanuel Luyirika, Mairead Murphy, Liz Namukwaya, Fatia Kiyange, Mackuline Atieno, Emilly Kemigisha–Ssali, Jenny Hunt, Kaly Snell, Scott A Murray, Mhoira Leng

**Affiliations:** 1 Global Health Academy, The Usher Institute of Population Health Sciences & Informatics, The University of Edinburgh, Edinburgh, Scotland, UK; 2 Makerere University, Kampala, Uganda; 3 African Palliative Care Association, Kampala, Uganda; 4 Bristol University, Bristol, UK; 5 Makerere University and Mulago Palliative Care Unit, Kampala, Uganda; 6 Independent Consultant, Lusaka, Zimbabwe; 7 University of Zambia, Lusaka, Zambia; 8 Primary Palliative Care Research Group, The Usher Institute of Population Health Sciences & Informatics, The University of Edinburgh, Edinburgh, Scotland, UK; 9 Cairdeas International Palliative Care Trust, Aberdeen, UK

## Abstract

**Background:**

The WHO is calling for the integration of palliative care in all health care settings globally.

**Methods:**

A 3.5–year program was implemented in 12 government hospitals, three each in Kenya, Rwanda, Uganda and Zambia. A four–pillared approach of advocacy, staff training, service delivery strengthening and international and regional partnership working was utilized. A baseline assessment was undertaken to ascertain needs, and 27 indicators were agreed to guide and evaluate the intervention. Data were also collected through surveys, interviews and focus groups.

**Results:**

Palliative care was integrated into all 12 hospital settings to various degrees through concurrent interventions of these four approaches. Overall, 218 advocacy activities were undertaken and 4153 community members attended awareness training. 781 staff were equipped with the skills and resources to cascade palliative care through their hospitals and into the community. Patients identified for palliative care increased by a factor of 2.7. All 12 hospitals had oral morphine available and consumption increased by a factor of 2.4 over two years. Twenty–two UK mentors contributed 750 volunteer days to support colleagues in each hospital transfer knowledge and skills.

**Conclusions:**

Integration of palliative care within different government health services in Africa can be achieved through agreed interventions being delivered concurrently. These include advocacy at Ministry, Provincial and District level, intensive and wide–ranging training, clinical and support services supported by resources, including essential medicines, and an investment in partnerships between hospital, district and community.

Globally, over 40 million people currently require palliative care annually [1]. This number will rise with the aging and increasingly multi–morbid population in all regions [2]. Low resource settings account for two–thirds of the global burden of disease [3,4], where 78% of those needing palliative care live, usually presenting with advanced disease [5].

As **Box 1** indicates, the integration of palliative care into health systems in Sub–Saharan Africa (SSA) faces challenges, including poverty, rising communicable and non–communicable diseases, fragile health systems, delayed health–seeking behaviors, poor transport networks, cultural beliefs, conflicts, and lack of access to essential medicines [6,7]. Few African countries have palliative care identified within their national health policies and strategies [8]. Only five countries have palliative care integrated in curriculums for health workers [9]. Governments want to provide adequate care for those with life–threatening illnesses, but lack persuasive evidence or funding to effectively deliver palliative care. Centres of palliative care excellence do exist, but their reach is limited, they are often not integrated within the health system and are reliant on external funding [10,11].

Box 1 **Research in context**

Evidence before this study:

- The WHO resolved in 2014 that integration was the only way to ensure universal access to palliative care. The Global Atlas of Palliative Care reveals limited integration and access in Africa.
- The need for palliative care services is strongly recognised within the UN Political Declaration on NCDs. However low resource countries prioritise prevention and cure over palliative care.
- There is little evidence with regard to “best practice” models for integrating palliative care in low resource settings, although several models of specialist palliative care services provide excellent care for a fortunate few.
  Added value of this study:
- This palliative care integration initiative was completed after the 2014 WHO resolution, putting us in a unique position to evaluate our programme in the context of the “building blocks” for integration highlighted in this resolution.
- Palliative care can be effectively integrated into different levels of government health systems in sub–Saharan Africa if several interventions are conducted concurrently.
- Advocacy, staff training, mentorship partnerships and improving delivery and supply chain for access to essential palliative care medicines and linkages can enable effective integration of palliative care into generalist healthcare.
- Each setting is unique but core components for integration remain.
- Resource–limited governments can successfully integrate palliative care with preventive and curative care but some funding is required for staff time, training, drugs.
- Utilising mentors from different settings enable mutual learning to take place, including from resource–limited to resource–rich settings.
- The four core intervention approaches utilised in this study should be tested in other national settings.
- This case study is used by the WHO in their guidance on how to integrate palliative care in resource poor countries.

Palliative care is on the global health agenda. The WHA resolution of 2014 directed member states to “*integrate evidence–based cost effective and equitable palliative care services in the continuum of care, across all levels of care*” [1]. To achieve Universal Health Coverage, a core component of Sustainable Development Goal (SDG) 3, palliative care is required [12].

A systematic review in 2011 found minimal evidence of effective models of palliative care [13]. An integrated service across all health service levels and settings may be effective in addressing the expanding need [4,14–18]. This paper sets out to provide evidence of impact through a large trial of the integration of palliative care within the health systems of Kenya, Uganda, Rwanda and Zambia.

## METHODS

A 3.5–year program of work jointly led by the University of Edinburgh Global Health Academy, the African Palliative Care Association (APCA) and Makerere University Palliative Care Unit (MPCU) was set up to build integrated models of palliative care provision in 12 hospitals in four countries. The lead partners worked through National Palliative Care Associations (PCA) or the Ministry of Health (MoH). Twelve hospitals, were chosen by the national partners. These included national referral hospitals, regional hospitals and district hospitals. The context of each of the countries is described in **Box 2**.

Box 2 **Context of the intervention**

The health systems of the four countries all differ but share characteristics such as low numbers of health workers, and village level health provision operated mainly by Community Health Workers who refer upwards to nurse led health care facilities offering treatment of common diseases, immunization and ante–natal care. They in turn refer to district hospital care through to regional then national tertiary–level referral services. In Uganda, Zambia and Kenya patients could self–refer through the system, but in Rwanda patients were required to pass through each level. In Zambia faith agencies provide an especially significant proportion of health care in addition to the government, in Kenya the devolved structure means health budgets are held at county level. Tertiary and national hospitals are complex, and carry a significant burden of disease but district hospitals offer a closer link with lower level health centers, and greater potential to access the enormous unmet need for palliative care in the community.

The program employed a health systems strengthening and capacity building approach, as advocated by the WHO [1,4]. The WHA Resolution called for member states to address the issues of policy, funding, supporting communities, training, supply of essential medicines, control of essential medicines, policy on essential medicines, partnership and the burden of non–communicable diseases [19]. Thus these issues, along with the six building blocks described in the WHO document “Monitoring the Building Blocks of Health Systems” helped to focus a four–pillared multi–layered approach of advocacy, staff capacity, service delivery and partnership, in the integration process (**Table 1**) [20].

**Table 1:** The four–pillared multi–layered approach adopted for integrating palliative care in health care

Advocacy	**Supported at three levels – country, hospital and community. A whole system approach was advocated for, including provision of palliative care in policy, access to medications, and inclusion in curricula and services.**
**Staff capacity**	A critical mass of generalist and specialist staff trained within each hospital network. Training included:
	***Basic training*** to a critical mass of staff of different cadres (supported by clinical placement modeling)
	***Advanced training*** on complex interventions and care strategies, included research and children’s palliative care
	***Training of trainers*** to cascade training to others
	***Specialist training*** for the future leaders of palliative care services. Where available, clinicians were sent for Diploma, Degree and Masters training
	***Community training*** raising awareness to community workers within the existing health system
**Service delivery**	Development of local policy, standards and protocols, improving and connecting patient pathways, referral systems and supporting frameworks for regular and reliable morphine procurement, provision and prescribing.
**Partnership**	Multi–layered partnerships including:
	-Partnerships between the lead organisations
	-Partnerships with the National Palliative Care Associations and the MOHs
	-Partnerships with each hospital
	Mentorship hubs – each hospital partnered with a small team of UK mentors who were experienced palliative care professionals, for guidance and support

An initial baseline was undertaken around each hospital to ascertain the nature and extent of palliative care provision. Twenty–seven key indicators were developed to guide the intervention and act as outcome measures (see Appendix S1 in **Online Supplementary Document(Online Supplementary Document)**). To build capacity within the existing systems, the 12 hospitals and their community catchment areas were supported to chart their vision for integrated palliative care. Hospitals remained responsible for all staffing costs while the program supported capacity building and training. During the intervention, quantitative and qualitative data were collected through surveys, pre and post training course questionnaires, interviews and focus groups and Most Significant Change methodologies [21]. A detailed final evaluation, utilizing both quantitative and qualitative data collection was undertaken and summarized in this paper and further in depth results will be reported elsewhere.

## RESULTS

Palliative care was integrated into each hospital in different ways. Rapid integration tended to occur where there was pre–existing exposure to palliative care. National level hospitals were slower to achieve integration because of their size and complexity. Results are reported according to the four intervention approaches.

### Pillar 1: advocacy

Advocacy achieved change at community, regional and national levels. Regionally a ‘Consensus Statement for Palliative Care Integration into Health Systems in Africa’ was adopted at the 2013 APCA hosted African Ministers’ meeting, committing to the ongoing integration of palliative care in each country represented [22]. Palliative care was included in the national plans of Kenya and Uganda and the National Health Strategic Plan in Zambia and Rwanda [23,24]. In Rwanda, the project directly led to palliative care being included in the Health Management Information System, national non–communicable diseases division planning, and to the adoption of national clinical guidelines disseminated to the hospitals.

Across the 12 hospital sites, 4153 community members attended advocacy awareness training through 73 events. These included sensitization for the hospital community and their referring hospitals and health centers, and training of Community Health Volunteers. Media coverage and community events, including burials and church services, increased coverage with Rwanda reporting 10 000 radio listeners supported by the national cellphone provider. Senior staff participation enhanced advocacy.

### Pillar 2: Building staff capacity through training and mentoring

By the end of the program, national partners were sustainably delivering training. Clinical placement sites were developed in each country providing centers of excellence to model best practice. 781 health professionals were trained in the 12 hospitals and their referring hospitals, of whom 520 also completed clinical placements where staff were mentored by clinical experts at in–country clinical placement sites (some already existing and others strengthened for the program) or the MPCU center of excellence in Uganda where all Rwanda trainees attended. A further 123 were trained as trainers. Additional requests from hospitals for training saw 39 staff trained in children’s palliative care, 81 in research skills and 60 in morphine procurement and prescribing (the latter as a national initiative in Rwanda). Training, utilizing a combination of classroom, ward sessions and clinical modeling, extended both depth and breadth of knowledge, establishing a critical mass of trained personnel with knowledge, skills and resources and also changed attitudes and values. Basic and advanced training took place across different cadres (**Table 2**).

**Table 2:** Numbers of health professionals trained

Training	Total	Cadre			
**Clinical – officer**	**Nurse**	**Doctor**	**Others**
Basic training: Introductory training in palliative care	614	43	391	49	131
Advanced training:					
-Research training	81	3	36	7	35
-Children’s palliative care	39	1	27	9	2
-Pharmacy training	34	0	0	0	34
Hospital Directors – management	26	0	0	0	26
Training of trainers	123	8	83	13	19
Specialist training; diploma/degree	36	2	27	4	3

Pre and post course questionnaires (immediate post training and six months post training) demonstrated that participants had improved their knowledge and skills, with improved performance and confidence. The final evaluation showed that training led to a step–change in clinical skills, including the ability to identify those requiring palliative care, to discuss with patients, and a better understanding of pain management. A trainee’s response of “*Pain is what the patient says it is*,” captured this shift; revealing how this commonly taught concept is becoming embedded in practice. Health workers described how this changed their clinical practice, by allowing patients to take extra doses of analgesia for breakthrough pain “without fear”. This included understanding children’s pain. A senior nurse explained of her staff:

“*Before they did not attend to children’s pain because they didn’t imagine they feel the level of pain that they do and they didn’t know how to score it. But now they manage pain even in children.”* (Nurse, Uganda)

Training created a change in mind set which practitioners considered, a *“life changing”* experience: “*before I thought it (palliative care) was about giving up*” explained one of the nurses from Kenya until she realized that palliation was active care. Training enabled staff to communicate better:

“*Now I have the heart of listening to the patients, talking, counselling and assessing them.” *(Nurse, Uganda)

Thirty–six health professionals undertook Palliative Care Diplomas and Degrees which gave staff increased status within their hospitals, and enabled staff to adopt a “whole systems approach” positioning them as palliative care leaders and advocates:

“*The training helped me to approach people in politics, or senior level. Before, the chief county officer would not take my phone calls. They have also recognised us as specialists. Now I am interested in the strategic plan.” *(Specialist trainee, Kenya)

“*Doctors have recognised that me and the others [specialist trainee colleagues] are experts in some PC areas… people ask us to do counselling, especially.” *(Nurse, Zambia)

Training was one component of building staff capacity, the other was on–site mentoring. Mentoring was provided by national partners, lead partners and overseas mentor colleagues. International mentors passed on their expertise through on–the–job training and clinical modeling. As a Kenyan health worker explained, this was essential for consolidating the clinical skills training:

“*KEHPCA [the Kenyan Palliative Care Association, who delivered the training course] gave us the theory, and mentorship gave us the practice.”* (Health worker, Kenya)

Palliative care teaching was embedded into pre–service, in–service and postgraduate health worker training institutions. The program successfully supported integration of palliative care into the undergraduate medical curriculum at the University of Zambia (UNZA), School of Medicine and the Faculty of Health Sciences at Moi University, Kenya. A Curriculum Toolkit: “*A practical guide to integrating palliative care into Health Professional Education*” (http://www.ed.ac.uk/global-health/research/project-profiles/health-systems-strengthening/thet/resources) was developed at the request of a country partner university, to give practical guidance and resources regarding the integration of palliative care core competencies into curriculums for health care workers.

### Pillar 3: service delivery

Improved outcomes were achieved across three core areas: improved identification of more patients for palliative care, development of management/referral systems, and morphine prescribing.

### Identification of patients for palliative care

**Figure 1:**
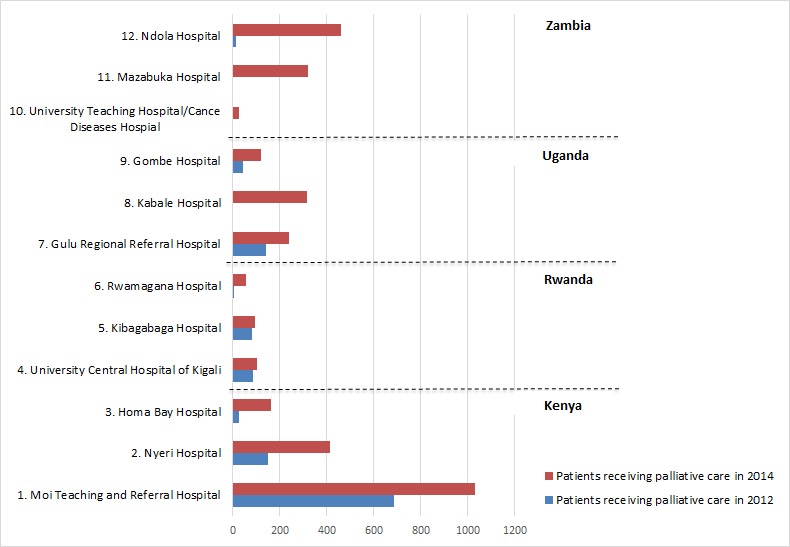
Number of patients using the palliative care services at the 12 project hospitals.

Overall, 2.7 times more patients were identified for palliative care, with some hospitals seeing an increase of 13 times and others formally identifying patients for the first time (**Figure 1** and **Table 3**).

**Table 3:** Patients identified for palliative care in the 12 hospitals by the Palliative Care Team

	Patients identified for palliative care		
**2012**	**2014**	**Ratio 2014/2012**
**Kenya:**			
1. MTRH	687	1030	
2. Nyeri Hospital	151	413	
3. Homa Bay Hospital	27	163	
***Total Kenya***	**865**	**1606**	**1.85**
**Rwanda:**			
4. CHUK	85	104	
5. Kibagabaga Hospital	80	92	
6. Rwamagana Hospital	4	56	
***Total Rwanda***	**169**	**252**	**1.5**
**Uganda:**			
7. Gulu Regional Referral Hospital	140	240	
8. Kabale Hospital	–	315	
9. Gombe Hospital	43	121	
***Total Uganda***	**183**	**676**	**3.7**
**Zambia:**			
10. UTH/CDH	*	26	
11. Mazabuka Hospital	*	319	
12. Ndola Central Hospital	11	462	
***Total Zambia***	**11**	**807**	**73.3**
**Total all countries**	**1228**	**3341**	**2.7**

*No formal palliative care team on site thus no patients recorded as being identified.

Many hospitals adopted a model of a named “link–nurse” in each ward or unit liaising with and supported by a specialist palliative care team. These nurses identified patients in their wards, and managed their care until their complexity required a more specialist input.

These systems of early identification became core to the integration of palliative care, preventing unnecessary medical interventions and starting discussions between patients, families and clinical staff on patient needs, goals and progress. They also facilitated integration, through development of an extended team and a referral network. One Kenyan health care worker commented:

*“Before I didn’t know how to liaise with the PC team. But then they gave us the mandate to be part of them. I could call them when I’m not able to handle some patients. The first time I needed to counsel a patient I called someone [from the core team] to assist me, then I did it myself, and then when someone asked me I did it with her.”* (Nutritionist, Kenya)

The researchers advocated for documentation of palliative care needs and services by nurses in case notes to become routine practice, so that patients receive appropriate and documented care. This remained challenging: at the end of the project 75% had information recorded (almost always by doctors) in their clinical records. In Rwanda a more advanced system is being trialled enabling health workers to write limited information in the notes such that there is a management plan within a national level template.

### Clinical management and referrals

All hospitals demonstrated improved policies and professional standards. The program contributed to 35 different strategies, standards, and protocols, including referral documentation, patient registers, assessment forms, clinical and audit protocols, national training materials and policies. Eleven new palliative care clinical protocols were adapted (from MPCU) and adopted as national documents in three countries, with adoption ongoing in the remaining country.

All hospitals developed stronger referral linkages, with central points for receiving and logging referrals. Some had a clear written referrals process. Most had a telephone referral system working alongside. An innovative approach was the establishing of a 24–hour telephone hotline at MTRH, Kenya, advertised on posters throughout the hospital for internal referrals, and given to patients on discharge.

### Morphine prescribing

Six of the 12 hospitals had no oral morphine available initially; all had it by the end. Morphine consumption and consistency of supply increased in all hospitals (**Table 4**). Specific prescribing training and continuing medical education sessions conducted by national partners, trainers, visiting mentors and by staff of the nascent palliative care teams broke down myths about morphine and empowered staff to prescribe and dispense morphine. A Ugandan nurse described how since the training morphine orders no longer expired on shelves but were prescribed by staff no longer fearful of morphine.

**Table 4:** Oral morphine consumption in hospitals

	Oral morphine consumption (mg)		Ratio 2014/2012
**2012**	**2014**	
**Kenya:**			
Moi Teaching and Referral Hospital	1 560 000	3 200 000	
Nyeri Hospital	40 000	220 000	
Homa Bay Hospital	0	66 500	
**Kenya total**	**1 600 000**	**3 486 500**	**2.2**
**Rwanda:**			
University Hospital of Kigali	0	77 800	
Kibagabaga Hospital	0	69 690	
Rwamagana Hospital	0	1920*	
**Rwanda total**	**0**	**149 410**	**–**
**Uganda:**			
Gulu Regional Referral Hospital	0	198 225	
Kabale Hospital	36 000	336 000	
Gombe Hospital	39 000	118 000	
**Uganda total**	**75 000**	**652 225**	**8.7**
**Zambia:**			
University Teaching Hospital/ Cancer Diseases Hospital	320 000	402 230	
Mazabuka Hospital (available from June 13)	0	37 915	
Ndola Central Hospital	5 200	93 340	
**Zambia total**	**325 200**	**533 485**	**1.6**
**Total countries**	**2 000 200**	**4 821 620**	**2.4**

In Rwanda, the program facilitated a national morphine framework meeting which resulted in the first national procurement agreements for morphine. The MoH requested further training for doctors and pharmacists, who then facilitated morphine prescription as a first–choice therapy for unrelenting pain. There was a shift in attitude and understanding in all countries. One doctor explained:

“*As of now, I have taught the group that there should be no pain in any patient. Now we are able to prescribe morphine*.” (Doctor, Kenya)

While managing morphine distribution, legislation and regulation was challenging but innovative strategies emerged:

“*They (pharmacists who have undergone training) could produce it and just give it to the other pharmacists. Or weigh it and send it to the other pharmacies. This would help a great deal – because I have come across patients who do not get morphine at the weekends.”* (Senior Doctor, Kenya).

The most remarkable change reported through the use of morphine was “silence”. Health workers in a number of hospitals poignantly said it was the absence of patients crying in pain on wards that spoke most powerfully of palliative care:

*“The staff are starting to understand that patients should not be screaming and crying in hospital!*” (Lead Nurse, Kenya)

Health workers reported that not only patients’ physical pain but also holistic pain was addressed and relieved,

“*Initially we didn’t know we were supposed to take care of pain for all of a patient’s life. As of now, I have taught the group that there should be no pain in any patient.”* (Doctor, Kenya)

*“I learnt about holistic care and finding out about what the patient needs….treating the patient as a person, not a case. Sometimes you feel you hit a brick wall, but PC taught me there is always something we can do.”* (Medical student, Zambia)

Staff were empowered to broach issues of death and dying and challenge cultural beliefs:

*“I understood PC in a holistic manner not just for dying patients but also the chronically ill. I understood that it meant social, spiritual and psychological support. I (myself) went and spoke in churches, and went with a local organisation to do screening and PC awareness at the churches. Right now I have a passion for PC.”* (Social Worker, Kenya)

*“At first people used to think perhaps you are cursing the patient to die to break bad news, now we are breaking bad news we realise it is not true.”* (Health Worker, Mazabuka)

Senior staff believed that the integration of palliative care empowered patients, allowing them to plan for the future. Staff described how integrated palliative care enhanced the whole care system. They spoke of no longer feeling helpless, because they could do something for patients and engage with families better. Their training helped them outside their work:

*“The training was fantastic – life–changing. Not only in the workplace, but also at home.”* (Pharmacist, Kenya)

### Pillar 4: partnership

Partnerships were developed at four levels. Regionally, hospital staff were supported to meet at national and international workshops and conferences on palliative care to present work and participate in specialist training. These included the Kenya and Uganda national palliative care conferences, the African Palliative Care Association conference, the International Multidisciplinary Pain Congress, the University of Edinburgh Global Palliative Care symposium, the International Conference on Advances in Palliative Care, and Pain and Patient Symptom Management.

At national level national partners, who were the lynchpin of project delivery and the main link between the steering group and the local hospitals, received advocacy training and funding both within national associations and within the government systems. In three countries the National Associations were a valuable asset to hospitals. In Rwanda the Ministry of Health took on the support role delivered by other country national associations At hospital level staff worked with local stakeholders, community volunteer workers, their referring hospitals and clinics. International mentors partnered with hospitals and national associations. Mentorship was delivered at different levels: 22 mentors from the UK visited hospitals, providing 621 on–site mentorship days and 145 days distance support; south–to–south mentorship was provided by the MPCU team and by National Associations and palliative care coordinators. Mentorship allowed staff to see new ways to use existing resources, while exchange visits to other facilities to see clinical practice stimulated quality improvement ideas. UK mentors spoke of reciprocal learning. For example, the major role of the family in the SSA context, the need for innovation in resource–constrained settings, and the impact of total integration into community hospitals, all provided models which mentors felt could influence UK care.

## DISCUSSION

The program helped establish systems of integrated care in 12 test sites across four countries. Multi–level advocacy raised the profile of palliative care and resulted in inclusion of palliative care in national plans, clinical guidelines, and health information systems. Training, modeling and mentoring established a workforce of generalist clinicians practising integrated palliative care. Morphine was more effectively procured and utilized in all hospitals and was often used as a step 2 medication in low dose (in keeping with common practice in Africa for a 2 Step approach). Systems to identify need and improve coverage and referral were created. Ownership of the program by each hospital and district health service bred success.

Narrative [11] and systematic reviews [13] have assessed the current degree of integration of palliative care in SSA [25]. This project went beyond mapping and model description into implementing integrated palliative care into national health systems [20]. We found that while weak health systems impede integration, integration can result in a stronger system as staff trained to provide palliative care are more motivated and provide holistic care, and greater staff, patient and family communication. National associations can have a key role and this was different in each of the 4 countries with particular impact from stable and well integrated associations. However there were challenges with funding and local politics. Government leadership and ownership of palliative care is essential.

The training program emphasized delivering outcomes rather than course content. This was achieved through mentors and local trainers prioritising embedding knowledge and skills into ongoing practice and supporting clinical placements. Negative beliefs about morphine, beliefs that curses are real causes of disease, and complex beliefs in the power of traditional medicine frequently existed [26]. These potential barriers were addressed and incorporated into training and sensitization talks with a wide range of staff, including mortuary attendants, and reception staff in the outpatient departments.

Identifying which patients might benefit from palliative care was a key and challenging component of training. We drew on two recent literature reviews [27,28], APCA guidance documents, a census study from Mulago Hospital, Uganda, and an evaluation of the MPCU model of link nurses to inform the scale up within the program [15,17,18]. Creating confidence in staff in all wards to identify patients who would benefit from palliative care greatly increased access. After staff training, patients received many aspects of palliative care support in the wards, and not necessarily referred to specialist palliative care services. Thus the number of patients receiving palliative care was probably under–documented. A previous study at Mulago Hospital Uganda has suggested that 75% of palliative care patients can have their care provided by trained ward teams without specialist referral [17]. Issues of quality and ongoing support pathways of care require further study.

It is more challenging to train and support generalists than to extend specialist palliative care through outreach work [11,29]. Integrated care has resulted in increased morphine prescription in faith–based hospitals in SSA [30]. We have importantly demonstrated this is also possible in government hospitals if Ministries of Health are committed to achieving integrated care, even with minimal resources.

Resource constraints in SSA mean that financing of palliative care is contentious. External financing for PC has been substantial but integrated sustainable national financing has been more difficult to establish [31]. This program significantly contributes to the financing debate for palliative care by effecting a low–cost integration of care into national systems and into national health plans so that funding can be identified [32]. One setting, Kibagabaga, showed significant cost savings by early pain control and shortening admissions [33]. This program suggests that advocacy and also national commitment to act are needed, alongside modest funding allocated within national and district health budgets.

### Strengths and limitations

This program was set up as a multi–country intervention to test integration strategies for palliative care in different locations and sizes of facilities. We did not have a control arm. We recognize that no change happens in isolation and the changes that this program brought about must be interpreted in the context of the dynamic health systems of each country. We did not collect health–related quality of life information directly from patients. We state the need to view the impact of this program in the context of the overall developments within each country, and to assess long–term outcomes.

### Implications

No one model of palliative care service provision fits every setting [25]. Innovations that embed palliative care values and integrate palliative care as part of good clinical practice within all specialties, cadres, and for all staff and for all diseases are indicated. The World Health Assembly resolution called for the Director General to “*encourage research on models of palliative care that are effective in low and middle–income countries, taking into consideration good practice*” [1]. This paper is one of the first programmes to report on integrating services in different hospitals in different countries in SSA. It provides a blueprint for integrated care provision throughout Africa. Specific recommendations which directly responds to the WHA Resolution are listed in **Table 5**.

**Table 5:** Recommendations

Governance and leadership	**Ministries of Health (MoH) need to integrate palliative care services in country policies, strategic plans and budgets. Ministries should drive this integration with a nominated person responsible for national palliative care who can work with all the stakeholders, including national associations and the various external donors and other development partners who frequently deliver standalone palliative care services.**
**Service delivery**	Patients with palliative care needs are found throughout all levels of the health care system. Therefore care should be integrated into each level (tertiary, secondary and primary levels) and across all life–threatening illnesses, with good referral networks for continuity of care. Clear service delivery protocols should be in place. Palliative care interventions should be based on the needs of patients and their families and not limited by disease or health care setting.
**Human Resources**	A critical mass of staff should be trained to understand and deliver a palliative care approach in all settings. Senior hospital staff should be included for effective integration. Both ongoing mentorship and modeling of palliative care are important to ensure the sustainability of services and enable the necessary behavior change in clinicians. The MoH should also ensure strategic deployment of palliative care trained staff with palliative care being incorporated into deployment planning, job descriptions, and training programmes.
**Finances**	This program showed that it is possible to integrate palliative care by utilizing existing staff and procurement systems. The main financial implications elate extra staff hours, staff capacity building for PC, mentorship and supervision all of which need to addressed in MoH budgets. The project also reveals that districts are willing to incorporate palliative care in their budgets once they obtain an understanding of the importance of the service.
**Medicine, vaccines and technology**	The MoH should ensure that palliative care essential medicines are on the country essential medicines list and that the necessary documentation and regulation are in place to make these medicines available and accessible to all who need them over 24 hours. They should also ensure that there is sufficient capacity for prescribing (by encouraging, for example, nurse prescribing) and resilient procurement processes with special attention to oral morphine.
**Strategic information**	The MoH should include palliative care in the Health Management Information Systems, such that all facilities are required and supported to report palliative care interventions. This should be wider than referral to specialist services. National level tools for data collection along with support and supervision will also be needed. The development of an evidence base which is contextual, high quality and value based should be resourced.

## CONCLUSIONS

Multi–level advocacy can facilitate country improved access to palliative care. Health systems need a convergence of national policies and regulations for promoting palliative care, continuous staff training and support and reliable procurement, availability, and access to, and use of, palliative care drugs in all areas all of which require long term funding. The concurrent advocacy, training, and improvements in service delivery and drug availability over the three years of this project did make a difference in the four countries studied. Further monitoring of the longer term benefits is required.

Good quality palliative care requires early detection of patients: long distances to health care facilities deters uptake, so engagement of all staff, clear referral pathways, and reliable links between community and hospital are needed. The approach to advanced life limiting illness and pain that is flexible, responsive, facilitative and creative can make a huge difference for patients, for providers and for communities. The WHO published in 2016 a practical manual on how to plan and implement palliative care services, integrated into existing health–care services, at national or subnational level. It contains some specific examples from this study and much useful guidance to make integration a reality in resource poor settings. (WHO 2016) (**Box 1**).

Each of the 12 participating hospitals exists as part of a referral system and activities were implemented across this wider system. A list of the participating hospitals is shown in **Table 6**.

**Table 6:** List of 12 participating hospitals

County	Level	Hospital	Beds
Kenya	National	Moi Teaching and Referral Hospital (MTRH)	800
Rwanda	National	Centre Hospitalier Universitaire Kigali (CHUK)	600
Zambia	National	University Teaching Hospital (UTH)	1600
Kenya	Regional	Nyeri County Teaching and Referral Hospital	300
Uganda	Regional	Gulu Regional Referral Hospital	300
Uganda	Regional	Kabale Regional Referral Hospital	250
Zambia	Regional	Ndola Central Hospital (NCH)	800
Kenya	District	Homa Bay Country Referral and Teaching Hospital	300
Rwanda	District	Rwanangama Hospital	220
Rwanda	District	Kibagabaga Hospital	230
Uganda	District	Gombe General Hospital	100
Zambia	District	Mazabuka District Hospital	160
