# Supplementary material for: Integrating palliative care into national health systems in Africa: a multi–country intervention study
Source: J Glob Health. 2017 Jun 9;7(1):010419. doi: 10.7189/jogh.07.010419 (PMC5475315; doi:10.7189/jogh.07.010419)
Supplement: Online Supplementary Document [file jogh-07-010419-s001.pdf]

Online Supplementary Document

Grant et al. Integrating palliative care into national health systems in Africa: a multi–country intervention study

J Glob Health 2017;7:010419

Appendix S1

**1**	Palliative care referral networks mapping showing stronger linkages at the end of programme as compared to at the start.	Service Delivery
**2**	Number of hospitals delivering an integrated community wide palliative care programme	Service Delivery
**3**	Number of hospitals modelling a public and primary health approach to palliative care	Service Delivery
**4**	Number of participating institutions demonstrating implementation of improved policies and professionals	Service Delivery
**5**	Percentage of institutional health strategies and professional standards/protocols which have been approved and signed off	Service Delivery
**6**	Number of institutional health strategies and professional standards/protocols to which the project has contributed to	Service Delivery
**7**	Number of clinical placement sites which have completed a standards audit and have a signed off quality improvement plan at the end of	Service Delivery
**8**	Number of baseline and end of project situational analyses completed	Service Delivery
**9**	Number of patients using palliative care services at 12 participating institutions	Service Delivery
**10**	Morphine consumption at the 12 hospitals	Service Delivery
**11**	Number of patients with a documented management plan of care	Service Delivery
**12**	Number of health workers demonstrating improved performance following training	Training
**13**	Number of health workers with skills to provide palliative care per 100,000 population	Training
**14**	Number of health workers demonstrating improved knowledge or skills after training	Training
**15**	Number of developing country health workers who participated in education / training	Training

**16**	Number of trained professionals completing clinical placements	Training
**17**	Number of Ministries of Health recognising palliative care in their national health plans	Advocacy
**18**	Number of advocacy / communication activities undertaken to influence the health agenda	Advocacy
**19**	Participating Associations influence development of Palliative Care in Africa	Partnerships
**20**	Number of UK volunteers demonstrating improved clinical and leadership skills	Partnerships
**21**	Number of new institutional health partnership MOUs in place	Partnerships
**22**	Number of UK health professional days spent volunteering overseas by end of programme	Partnerships
**23**	Number of UK health professional days spent providing remote support to overseas partners	Partnerships
**24**	Number of community people attending awareness training.	Advocacy
**25**	Number of community health awareness or mobilisation campaigns	Advocacy
**26**	Number of health professionals applying their skills and learning to	Training
**27**	Number trained as trainers	Training
